# Toxic Effect of Metal Doping on Diatoms as Probed by Broadband Terahertz Time-Domain Spectroscopy

**DOI:** 10.3390/molecules27185897

**Published:** 2022-09-11

**Authors:** Rohit Kumar, Melania Paturzo, Angela Sardo, Ida Orefice, Qiucheng Yu, Andrea Rubano, Domenico Paparo

**Affiliations:** 1 Physics Department ‘E. Pancini’, University ‘Federico II’, 80126 Naples, Italy; 2 CNR-ISASI, Institute of Applied Sciences and Intelligent Systems ‘E. Caianiello’, 80078 Pozzuoli, Italy; 3 Marine Biotechnology Department, Zoological Station ‘A. Dohrn’ (SZN), 80121 Naples, Italy

**Keywords:** broadband THz-TDS spectroscopy, diatoms, heavy metal pollution, marine ecotoxicology, bioremediation, solvating water

## Abstract

The global marine environment is increasingly affected by human activities causing climate change, eutrophication, and pollution. These factors influence the metabolic mechanisms of phytoplankton species, such as diatoms. Among other pollutant agents, heavy metals can have dramatic effects on diatom viability. Detailed knowledge of the interaction of diatoms with metals is essential from both a fundamental and applicative point of view. To this aim, we assess terahertz time-domain spectroscopy as a tool for sensing the diatoms in aqueous systems which mimic their natural environment. Despite the strong absorption of terahertz radiation in water, we show that diatoms can be sensed by probing the water absorption enhancement in the terahertz range caused by the water–diatom interaction. We reveal that the addition of metal dopants affects this absorption enhancement, thus enabling the monitoring of the toxic effects of metals on diatoms using terahertz spectroscopy. We demonstrate that this technique can detect the detrimental effects of heavy metals earlier than conventional methods such as microscopy, enzymatic assays, and molecular analyses aimed at assessing the overexpression of genes involved in the heavy metal-stress response.

## 1. Introduction

The global marine environment is increasingly affected by human activities that cause climate change, eutrophication, and pollution [1]. The latter has a strong impact on aquatic environments and living organisms residing there. Among pollutants, heavy metals represent a serious problem since they are nondegradable elements that accumulate in water and soils, and are widely present in several industrial, civil, and agricultural discharges [2,3]. Some of them are considered essential elements for their biological roles [4,5] but they can become toxic at high concentrations causing impairments in cell metabolism [6]. Heavy metals can significantly affect microalgal physiological states, causing alterations in silicon metabolism [7], damage to the photosynthetic apparatus [8,9,10], changes in cell and organelles morphology [11,12], and oxidative stress [13,14,15]. Considering that microalgae are the basis of the aquatic trophic network, reduction in their growth rates or the loss of species that are more sensitive to heavy metals can seriously affect aquatic ecosystems and alter biodiversity. Moreover, the accumulation of heavy metals dramatically increases the risk of biomagnification in fish and filter feeders [16,17]. In this work, we investigated the effects of different copper concentrations on a diatom species. We chose this metal since it is present in fertilisers, pesticides, antifouling paints, and several industrial discharges [18].

The toxic mechanism of copper is rather complex with many factors which can interfere, such as the variations in nutrient stoichiometry. It has been deeply investigated using several techniques, including spectroscopic tools [19]. However, many aspects of the interaction between metals and diatoms are still poorly understood. This is also due to the lack of suitable spectroscopic techniques to add complementary information to those provided by more standard techniques. On the other hand, a better comprehension of the adsorption of metals on diatoms may help in designing more efficient biosensors for water pollution monitoring [20,21,22,23]. As biosensors for metal pollution, the standard methodologies are based on the identification of diatoms with specific shapes and frustule ornaments [24,25]. However, these techniques are time-consuming and usually do not take into account the specific distribution of species in different regions. In this respect, new spectroscopic tools may provide faster and more precise screening capabilities.

Optical vibrational spectroscopies, such as Raman spectroscopy and infrared absorption spectroscopy (IAS), have proven to be valuable tools for the study of microscopic algae since they are label-free and can also be used in situ [26]. Raman and IAS spectroscopies are complementary instruments because they probe different molecular vibrational modes, excited by one- or two-photon processes in the case of IAS and Raman spectroscopy, respectively. Raman spectroscopy holds an important drawback: the generally low signal can be obscured by the diatom autofluorescence. This leads to the use of intense visible pump light which can dramatically affect diatoms. This issue is partially absent in the IAS, but there are other limitations that innovative techniques may circumvent. For instance, an intriguing effect that has been investigated by the IAS is a structural reorganization of the biomass induced by the water-mediated diatom–diatom interactions (the so-called ‘matrix’ effect) [27]. Incidentally, this effect biases the IAS spectra to the detriment of an accurate determination of the biomass content. This phenomenon may be related to a structural reorganization of the hydrogen-bond (HB) network in which diatoms are immersed. The IAS spectroscopy can provide only indirect information on this global reorganization since it only probes specific ‘local’ bond vibrations.

Terahertz spectroscopy may be considered an extension of IAS to the extreme range of the THz frequencies. This frequency interval has long been known as the ‘THz gap’ due to the difficulty of having efficient sources and detectors for this elusive radiation. However, thanks to the advances in laser technology over the past two decades, it is now possible to have THz table-top spectrometers pumped by intense and broadband THz pulses [28,29]. The use of intense beams allows a significant transmission of the THz light through aqueous samples over a wide range of THz frequencies [30]. The terahertz spectroscopy based on the use of such THz pulses is universally known as THz time domain spectroscopy (THz-TDS) [31]. In recent years, THz-TDS has proven to be an invaluable complementary vibrational probe for investigating in vivo biosystems since, given the low energy of the THz photons, it is not harmful to living cells. THz-TDS has been used to probe global vibrations and structural deformations in biomolecules, or the collective vibrations of the HB network of the water molecules [32]. These excitations do not lay in the infrared spectral range and hence they cannot be directly probed by Raman spectroscopy or IAS. Therefore, THz-TDS displays two fundamental advantages: (i) it is much less harmful to in vivo diatoms; (ii) it can directly probe the global structural reorganization of the HB network hosting the diatoms and the macromolecular vibrational modes of the diatoms themselves. The latter feature is of particular interest for investigating the above-mentioned ‘matrix’ effect. In Table 1 we synthesize the main characteristics, strengths, and drawbacks of these techniques.

**Figure 1:**
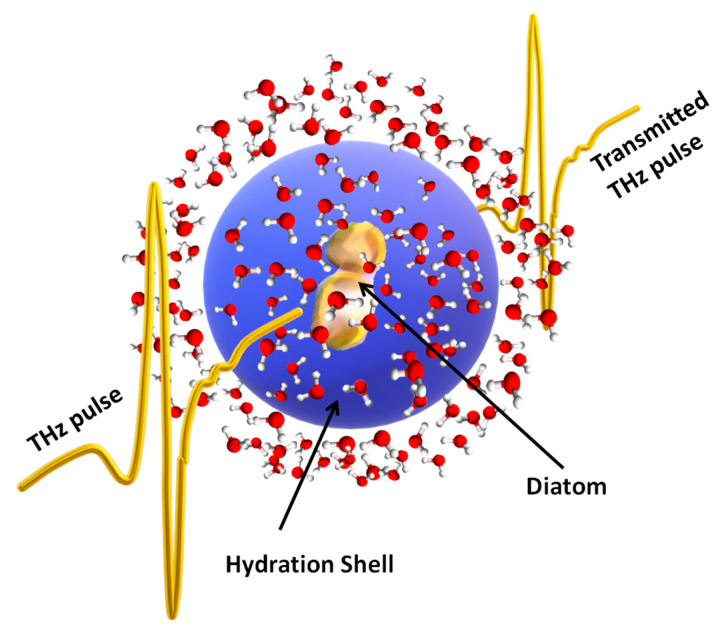
The principle of THz-TDS: a sub-picosecond THz pulse passes through the sample, here formed of diatoms in water. The transmitted pulse is attenuated and delayed because of the absorption coefficient and the refractive index of the sample. Therefore, it carries information on these two parameters at all the frequencies composing the pulse bandwidth.

To assess THz-TDS as a complementary spectroscopic tool for investigating diatoms we applied this technique to the diatom species *Skeletonema pseudocostatum*. We measured the THz absorption spectra of the latter in a seawater-based culture medium (SBCM) and at different concentrations of copper, as a heavy metal pollutant. *Skeletonema* belongs to the centric diatoms having multiradial valve symmetry and is mainly found in shallow waters [33]. These diatoms are thus suitable as pollution indicators of coastal environments. As explained in more detail in Section 4.2., we used a THz spectrometer that was built by the authors in which fast and intense single-cycle THz pulses were generated starting from intense laser pulses [34]. The details of this homemade setup are presented in Section 4. Notwithstanding the technological challenge, the concept behind THz-TDS is straightforward and it is schematically shown in Figure 1. A THz pulse with a duration of hundreds of femtoseconds, hence carrying a wide range of THz frequencies, passed through a sample (diatoms in water in the figure). The transmitted THz pulse was then measured as described in Section 4.2. We note that in THz-TDS experiments the time waveform of the optical THz field (amplitude and phase) was measured, not only its intensity. Therefore, the measured pulse carried information on both the refractive index, n(ν), where ν was the frequency, and the absorption coefficient, α(ν), of the sample. This information can be extracted with a suitable numerical procedure based on a fast Fourier transform, thus providing the full α(ν), and n(ν) spectra of the material under investigation over the whole range of frequencies composing the pulse bandwidth, without approximated numerical methods, such as Kramers–Kronig relationships, which are often used in the IAS [35].

## 2. Results

**Figure 2:**
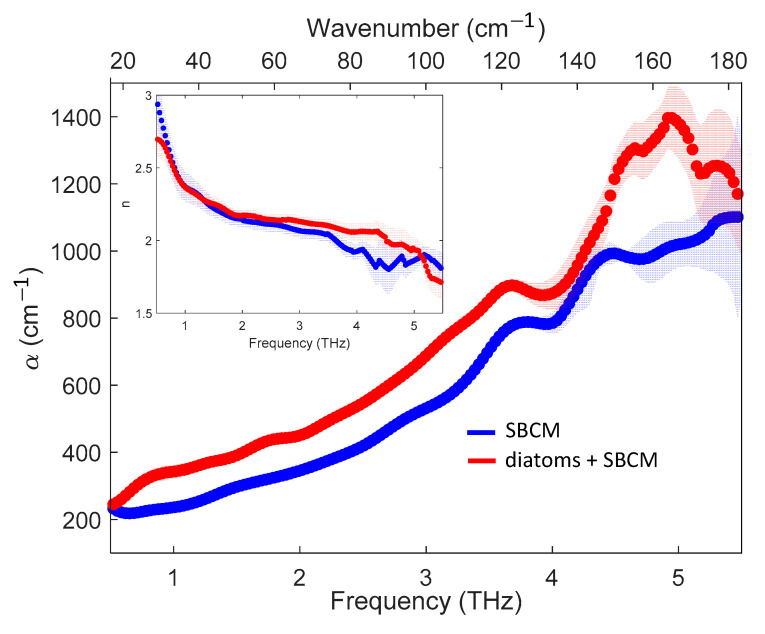
The THz absorption spectra of SBCM (blue curve) and diatoms in SBCM (red curve). In the inset, the corresponding refractive indexes are shown. The shaded areas indicate the measurement statistical error with a confidence level of 66% (one σ). The shaded areas overlap with the marker size up to 3.5 THz. On the upper *x*-axis we report the light wavenumber in cm^−^^1^.

In the main panel of Figure 2, the THz absorption spectra of SBCM (blue curve) and SBCM with diatoms (red curve) are reported. We indicated these samples as ‘baseline’ and ‘control’ samples, respectively. In the inset of the figure, the corresponding refractive indices are shown too. The faded areas represent the measurement of statistical errors at a confidence level of 66% (one σ). It is worth noting that the curves became more oscillating and the shaded areas larger at the highest frequencies due to an overall decrease in the signal-to-noise ratio. These oscillations introduced peaked structures in the absorption spectra that were unphysical for liquid samples. Therefore, in the highest frequency portion of the spectra, we applied a spatially variant moving average filter, analogous to the one proposed by Pupeza et al. [36].

In the ‘control’ sample we observed an overall enhancement of the absorption coefficient (absorption ‘excess’) compared to that of the ‘baseline’ over the entire frequency range, with a notable exception around 4.2 THz, where the two graphs overlapped. Above 5 THz the two curves displayed strong fluctuations and wider shaded areas. Therefore, we focussed our attention on the spectra from 0.5 to 5 THz only. In this range, the absorption increase was extraordinarily strong over the whole frequency range. As an example, at 2 THz, we had an absorption ‘excess’ of about 30%. The corresponding refractive index spectra almost overlapped over the whole frequency range with small deviations that were nevertheless within the error bars. These results clearly demonstrated that the THz absorption spectra are sensitive to the presence of diatoms in SBCM, while the refractive index spectra are not. The results reported in Figure 2 unambiguously show that THz absorption spectroscopy can be used for probing diatoms in aqueous environments. Therefore, in the following, we will focus our attention mainly on the absorption spectra. We note that the initial cell concentration (900,000 cells/mL) used in this work is much higher than those found in nature, but the main goal of this work is to validate a new methodology to detect diatoms and the copper-induced effects on them in real-time, rather than mimicking what happens in their natural environments. However, the large absorption enhancement we have measured showed that detecting lower densities of diatoms is highly feasible. This will be assessed in future experiments. Moreover, the ‘matrix’ effect mentioned before appears at sufficiently high concentrations. Therefore, the use of high concentrations allows us to find possible signatures of this effect in the THz spectra.

**Figure 3:**
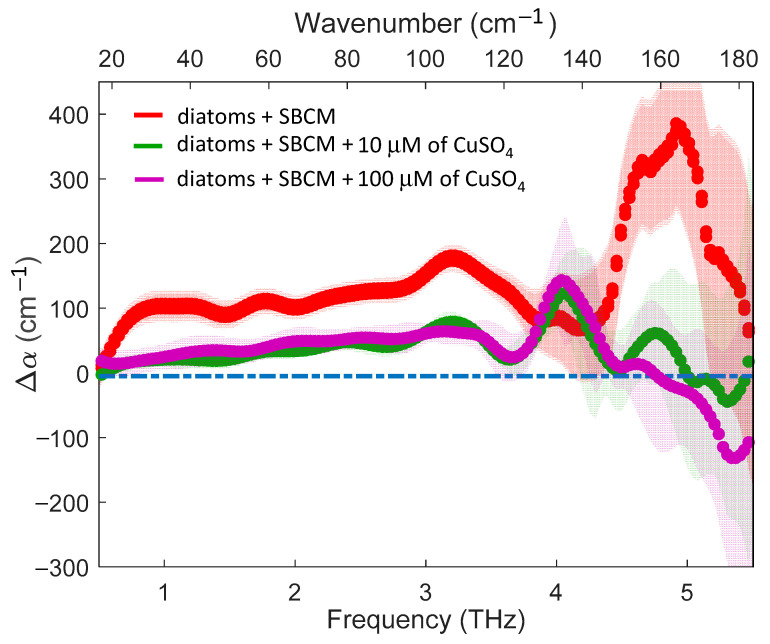
THz absorption variation as compared to the ‘baseline’ spectrum for diatom in SBCM, undoped (red line) and doped (green and purple lines). The dot-dashed blue line represents the zero level.

Now, let us turn our attention to the variation of the absorption spectra when the samples are doped with pentahydrate copper sulfate (CuSO_4_). In this study, we used two different concentrations of copper: 10 and 100 µM. To compare the different samples, we introduced a new parameter that measures the absorption variation compared to the ‘baseline’ spectrum, i.e., $\Delta \alpha =\alpha_{sample}-\alpha_{baseline}$, where sample stands for ‘control’, ‘10 μM’, or ‘100 μM’. A sample displays an absorption ‘excess’ or ‘defect’ when Δα is positive or negative, respectively. The absorption variation Δα for the three samples is shown in Figure 3. In accordance with the previous observation, the ‘control’ sample demonstrated an absorption ‘excess’ over almost the whole frequency range. Although in a range where fluctuations became stronger, we observed a significant peak of Δα around 5 THz. In both samples doped with copper there was an evident reduction of Δα compared to the ‘control’ sample over the full frequency range, except for the region around 4.2 THz where the three curves almost overlapped with the zero level (blue dashed line). The graphs of Δα for the two concentrations strongly overlapped, indicating that starting from a concentration of 10 μM the effect of the metal doping on the reduction of the absorption ‘excess’ had already saturated. In future research, it would be interesting to use lower concentrations to verify the existence of a trend in the reduction of Δα as a function of the concentration.

## 3. Discussion

The first result of this work to highlight was the capacity of THz-TDS in sensing the presence of diatoms in aqueous solutions. To the best of our knowledge, this is the first application of THz-TDS to the study of living diatoms in an aqueous complex medium, mimicking their natural environment. This was not granted from the beginning since it is usually very difficult to extract information on solutes dissolved in aqueous solutions because of the strong absorption of water in the THz range. To have a quantitative idea of this it is sufficient to consider that the absorption coefficient of most dried biomolecules is ∼10 cm^−1^ at 1 THz against ∼200 cm^−1^ of that of the liquid water at the same frequency. Therefore, an interesting question arises here: what is the origin of the absorption ‘excess’ observed in Figure 2?

If water and its solute do not interact, the THz spectrum of the solution simply retraces that of pure water with an overall reduction of the absorption coefficient due to the replacement of water volume by the much less absorptive solute (‘volume-displacement’ effect). This absorption ‘defect’ becomes larger as the solute concentration increases. In contrast with this simple picture, a lot of research on proteins in water has instead observed an ‘excess’ of the absorption in the THz range [37,38]. This effect has been ascribed to the formation of a ‘hydration shell’ around the solute that may extend for several molecular layers (see Figure 1). The water molecules in this ‘hydration shell’ behave differently from those in the bulk, displaying a dynamic that may be both faster or slower than that in the bulk. This dynamic strongly depends on the nature of the solute–water interaction and hence, under some circumstances, the ‘hydration shell’ can become an indirect probe of the solute behaviour [38].

In our case, things were much more complex. First, compared to the above-mentioned works, the observed absorption enhancement was very large. This is certainly related to the complexity of our samples made of unicellular microorganisms that are orders of magnitude bigger than single proteins. In particular, diatoms have intracellular organelles and compartments where water molecules can be strongly confined. These water molecules also behave differently from bulk water similar to the molecules residing in the hydration shell [39,40,41]. Second, we must consider the aforementioned ‘matrix’ effect. In this case, the diatom–diatom interactions mediated by the water molecules can lead to a structural reorganization of the HB network with a consequent enhancement of specific collective vibrational modes in the THz range.

In literature, all these forms of water that in biosystems behave differently from the bulk water are named ‘biological water’ [41]. At this stage, we are not able to weigh the specific contribution of all these effects, i.e., hydration shell, confined water, or ‘matrix’ water. However, it is clear that only the presence in our system of ‘biological water’ could explain the observed absorption ‘excess’. The latter is strongly influenced by the water–diatom and diatom–diatom interactions. Therefore, our THz absorption spectra carried information on these interactions and hence on the diatom viability.

When we added copper to our solutions, we observed a strong reduction of the absorption excess over the entire frequency range, with a remarkable residual enhancement of the absorption around 4 THz. First, we noted that the addition of the copper sulfate could not directly affect the spectra of SBCM in terms of volume displacement, given the low concentrations used here, the small ion volume, and the low copper molar attenuation [42].The latter consideration leads us to conclude that the observed reduction of Δα in doped samples could be ascribed to the effect of metal doping on the diatoms with a consequent disturbance of the ‘biological water’ in the doped samples. Therefore, a few hours after doping our samples with copper, we could already measure a dramatic modification of diatom viability. It is interesting to compare this result with the estimate of the number of living diatoms as measured by the Bürker chamber method [43]. In the first hours after the THz-TDS experiment till the day after, no significant variations in the number of living diatoms could be measured. Therefore, our results clearly demonstrated the ability of the THz-TDS in detecting the structural changes that had occurred already in diatoms that were still alive but whose functionality had started to be affected by the poisoning action of the metals. It should be noted that we started to record the first spectrum a few hours after the sample preparation because of logistical reasons (the sample preparation laboratory and the THz apparatus are not in the same building). However, as explained in Section 4.2., a single measurement took less than one minute and hence, in principle, the diatom viability might be monitored almost in real-time. We will explore this possibility in a future experiment.

Despite the large statistical fluctuations at higher frequencies, we observed that in the portion of the spectrum above 4.5 THz, Δα was almost zero, thus here the spectra of doped samples perfectly overlapped with that of SBCM. This must be compared with the peak of Δα observed in the ‘control’ sample. We highlighted that in pure water this part of the spectrum was largely influenced by the HB stretching modes [44]. Therefore, our results suggest that the diatoms poisoned by the metal were no more able to maintain the HB structure with a consequent loss of the spectral weight associated with these vibrational modes. This point deserves to be explored more in the future from both an experimental point of view, by increasing the signal-to-noise ratio at the highest frequencies, and from a theoretical point of view to single out the contributions to the THz spectrum of specific collective vibrational modes.

## 4. Materials and Methods

### 4.1. Sample Preparation

The marine diatoms *Skeletonema pseudocostatum* were isolated with the capillary pipette method [45] from the Sarno River mouth (40.72875 N, 14.466432 E, Naples, Italy), and identified by 18s and 28s rDNA genes, which have been successfully used in previous works to characterise analogous species belonging to this genus [46,47,48]. The microalgae (initial concentration: 900,000 cells/mL) were maintained in an f/2 medium [49] prepared with sterile, filtered (0.22 µm), natural seawater collected from the oligotrophic areas of the Gulf of Naples. The diatoms were exposed to 0 (‘control’ sample), 10, or 100 µM of copper. In the latter two cases, the copper concentration was obtained by dissolving appropriate amounts of copper sulfate pentahydrate in the culture medium.

The concentration of living diatoms in all the samples was estimated by cell counting with a Bürker chamber [43]. Each experiment was performed in triplicate. Ten mL of each sample were taken immediately after adding metals in the culture medium for measuring the THz absorption spectra as explained in the next subsections. A 10 mL aliquot of the culture medium was employed as a baseline for the THz measurements.

### 4.2. TDS-THz Experiment

**Figure 4:**
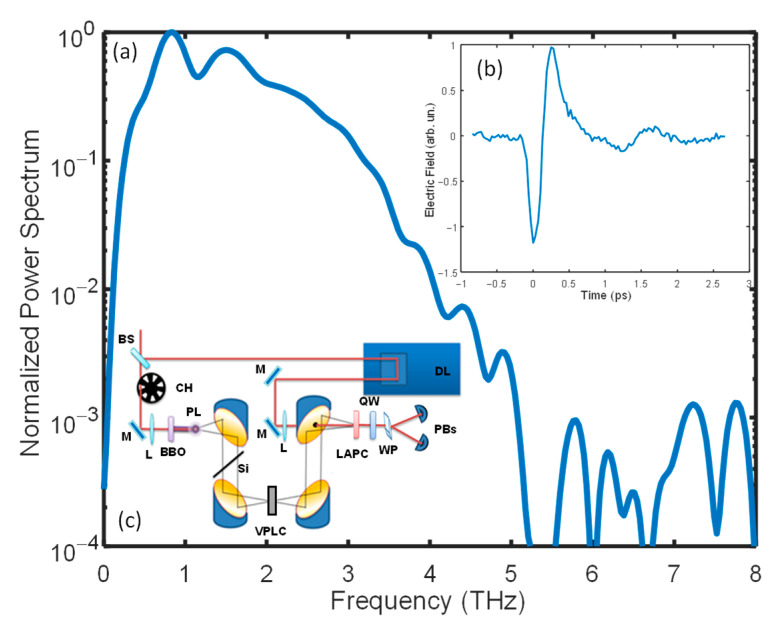
Panel (a) shows an example of the power spectrum of a THz pulse propagating in nitrogen, transmitted through the most absorbing sample, and detected by electro-optic sampling in LAPC (reported in the logarithm scale). Note that the signal is up to 5 THz greater than the background noise. In panel (b) the corresponding THz pulse is displayed. In panel (c) the scheme of our THz spectrometer is shown: BS, beam-splitter; CH, triggered chopper; L, lens; BBO, SHG crystal; PL, plasma; Si, silicon wafer; M, mirror; DL, delay line; VPLC, variable-path liquid cell; LAPC, electro-optic crystal; QW, quarter-waveplate; WP, Wollaston prism; BPs, balanced photodiodes.

The laser pulses used for THz generation were provided by a regeneratively amplified femtosecond laser (Coherent Legend), which delivered optical pulses of 35 fs of duration. The central wavelength of the laser was ∼800 nm and the bandwidth (full width at half-maximum) was ∼80 nm. The main part of the laser beam passed through the beam-splitter, as shown in the diagram of our THz spectrometer in Figure 4c, and was focused by a lens. After the lens, the beam passed through a β-barium borate nonlinear crystal that converted part of the fundamental beam into its second harmonic. At the focal point, these intense beams generated a plasma formed by ionized air. Here, broadband and ultrashort THz pulses were generated via four-wave mixing of the fundamental and second-harmonic photons [34]. The THz bandwidth was limited only by the laser bandwidth and therefore the highest generated frequency was 40 THz. The much less intense beam reflected by the beam-splitter was sent to an adjustable delay line and used for probing in time the generated THz pulse using the electro-optic effect in a suitable nonlinear crystal [35]. The latter technique allowed a coherent detection of the THz electric field (amplitude and phase). In the present work, we used a nonstandard crystal to reach efficient detection up to 10 THz: LAPC [50]. The spectrometer was closed in a chamber filled with high-purity (99.999%) nitrogen to strongly reduce the signal absorption by humid air. In Figure 4a an example of the power spectrum of a THz pulse propagating in nitrogen, transmitted through the most absorbing sample, and detected by electro-optic sampling in LAPC, is reported in logarithm scale. In Figure 4b the corresponding time waveform of the optical field is shown. Despite the bandwidth of our detection crystal, the sample absorption drastically reduced the transmitted signal, so that the signal-to-noise ratio for the experiments was good only up to about 5 THz in the most absorbing samples. This bandwidth, however, is not usual in THz-TDS experiments on aqueous solutions. The liquid samples were placed in a self-assembled variable-path cell with silicon access windows which had a good and flat transmission up to 20 THz. The cell path could be continuously varied from 1 mm down to a few microns with an average step of about 14 µm. For all measurements, we used the following thicknesses: 22, 29, 47, 64, 79, and 94 µm. For each sample, a measurement for all thicknesses took 15 min. We note that a single measurement lasted less than one minute. The remaining time was needed to manually vary the cell path. Therefore, the 15 min may be heavily reduced by implementing a motorized stage to vary the thickness. This improvement would allow us to perform measurements almost in real-time.

### 4.3. Absorption Coefficient and Refractive Index Extraction

The THz waveforms *E(t)* measured for different thicknesses were transformed in the frequency domain by fast Fourier transform to obtain the complex field $\tilde{E}\left(\nu \right)=E\left(\nu \right)e^{i\phi \left(\nu \right)}$, where E(ν) and ϕ(ν) represent the module and the phase of the complex field, respectively. As we will see in the following, these two quantities were directly linked to the complex refractive index, $\tilde{n}\left(\nu \right)=n\left(\nu \right)+ik\left(\nu \right)$, of the sample. Here, k is the extinction coefficient. The latter is related to the absorption coefficient by α=4πνk/c, with c the speed of light in vacuum. In principle, the ratio of the Fourier transforms of only two THz waveforms with and without the sample, with the first trace used as a reference, was sufficient to provide the frequency-dependent complex refractive index $\tilde{n}\left(\nu \right)$. However, the container windows complicated this procedure. Therefore, the complex refractive index was more reliably extracted by isolating the sample response from the window response through measurements at varying sample thicknesses d. In detail, the complex refractive index could be obtained by using the following Beer–Lambert generalized relationships [39]:

(1) $$n\left(\nu \right)=c\;\frac{\phi \left(\nu ,d+\Delta d\right)-\phi \left(\nu ,d\right)}{2\pi \nu \Delta d}=\frac{c\Delta \phi \left(\nu \right)}{2\pi \nu \Delta d}$$

(2) $$k\left(\nu \right)=c\;\frac{\ln E\left(\nu ,d\right)-\ln E\left(\nu ,d+\Delta d\right)}{2\pi \nu \Delta d}=\frac{c\Delta \ln E\left(\nu \right)}{2\pi \nu \Delta d}$$

For all our measurements *d* = 22 µm, i.e., the thickness of the thinnest sample, while Δd was the change in thickness. As it is apparent from Equations (1) and (2), the phase change Δϕ(ν) and the change of the natural logarithm of the electric field module ΔlnE(ν) depended linearly on Δd for a given frequency. Therefore, at each frequency, a linear model could be used for fitting the experimental results to extract n(ν) and k(ν), which were the slopes of these linear models. To fit the latter, we used a standard ‘chi-square’ minimization with the axis intercept of the linear model constrained to zero [51].

In conclusion, we note that Equations (1) and (2) were valid only if multiple reflections in the thin sample layer were neglected (those occurring in the silicon windows were not considered since they appear out of the measurement temporal window). In the case of thin samples this was possible only for very absorbing samples, as in our case, since the pulse was strongly attenuated during the back-and-forth trip so that the echoes of the main pulse became negligible.

## 5. Conclusions

We have demonstrated the ability of THz-TDS in sensing the diatoms in aqueous model systems. Due to the strong absorption of water in the THz range, the spectral signatures of diatoms could not be detected directly, but we found their footprints in the water absorption variation due to the water–diatom interactions. We observed an ‘excess’ of absorption over the entire interval of the THz frequencies.

This result allowed us to widen the set of spectroscopic tools for investigating marine organisms with instruments that provide complementary key information. In this respect, compared to the Raman and IAS spectroscopies, the THz-TDS may probe the large-scale vibrations of diatoms and the hydrogen-bond network of water and hence provide information on their global structural reorganization.

When metal dopants were added, the ‘absorption’ excess was strongly reduced over the entire THz frequency range. This points to a strong structural change of the ‘biological water’ acting in the doped samples. These absorption variations could be used for probing the biocide effect of metals on diatoms. We note that these changes were already significant a few hours after the doping when the presence of dead diatoms was very low or completely absent. This demonstrates that THz-TDS is suitable to monitor the poisoning effect at the earliest stage when the diatoms are still alive but have started to become unhealthy. Finally, we note that because of logistical reasons we were unable to monitor these effects just a few minutes after the doping, while, in principle, the technique has a time resolution of only a few minutes.

## Figures and Tables

**Table 1:** Comparison of the main characteristics, strengths, and drawbacks of the different techniques. Note that one- or two-photon processes probe vibrational excitations with different symmetries. In this respect, Raman spectroscopy is complementary to IAS and THz-TDS.

Technique	Optical Process	Vibrational Mode	Real-Time Capability	Harmful	Water Absorption
Raman	two-photon	single-bond	YES	YES	LOW
IAS	one-photon	single-bond	YES	LOW	MEDIUM
THz-TDS	one-photon	Collective	YES	NO	STRONG

## Data Availability

The datasets generated during or analysed throughout this study are available from the corresponding author upon reasonable request.
